# Extracellular l-arginine Enhances Relaxations Induced by Opening of Calcium-Activated SKCa Channels in Porcine Retinal Arteriole

**DOI:** 10.3390/ijms20082032

**Published:** 2019-04-25

**Authors:** Ulf Simonsen, Anna K. Winther, Aida Oliván-Viguera, Simon Comerma-Steffensen, Ralf Köhler, Toke Bek

**Affiliations:** 1 Department of Biomedicine, Pulmonary and Cardiovascular Pharmacology, Aarhus University, Wilhelm Meyers Allé 4, DK-8000 Aarhus C, Denmark; annawinther@chem.au.dk (A.K.W.); simoncomerma@biomed.au.dk (S.C.-S.); 2 BESICoS group, Aragón Institute of Engineering Research, IIS-Aragón, University of Zaragoza, 50009 Zaragoza, Spain; aidaolivanviguera@gmail.com; 3 Aragón Agency for Research and Development (ARAID) at IACS and IIS Aragón, 50009 Zaragoza, Spain; kohler@araid.es; 4 Department of Ophthalmology, Aarhus University Hospital, DK-8000 Aarhus C, Denmark; toke.bek@mail.tele.dk

**Keywords:** NO synthase, calcium-activated potassium channel, cationic amino acid transporter, relaxation, retinal arterioles, porcine

## Abstract

We investigated whether the substrate for nitric oxide (NO) production, extracellular l-arginine, contributes to relaxations induced by activating small (SKCa) conductance Ca^2+^-activated potassium channels. In endothelial cells, acetylcholine increased ^3^H-l-arginine uptake, while blocking the SKCa and the intermediate (IKCa) conductance Ca^2+^-activated potassium channels reduced l-arginine uptake. A blocker of the y+ transporter system, l-lysine also blocked ^3^H-l-arginine uptake. Immunostaining showed co-localization of endothelial NO synthase (eNOS), SKCa3, and the cationic amino acid transporter (CAT-1) protein of the y+ transporter system in the endothelium. An opener of SKCa channels, cyclohexyl-[2-(3,5-dimethyl-pyrazol-1-yl)-6-methyl-pyrimidin-4-yl]-amine (CyPPA) induced large currents in endothelial cells, and concentration-dependently relaxed porcine retinal arterioles. In the presence of l-arginine, concentration-response curves for CyPPA were leftward shifted, an effect unaltered in the presence of low sodium, but blocked by l-lysine in the retinal arterioles. Our findings suggest that SKCa channel activity regulates l-arginine uptake through the y+ transporter system, and we propose that in vasculature affected by endothelial dysfunction, l-arginine administration requires the targeting of additional mechanisms such as SKCa channels to restore endothelium-dependent vasodilatation.

## 1. Introduction

Disturbances in retinal blood flow are involved in the pathophysiology of major sight-threatening diseases, such as age-related macular degeneration, primary open angle glaucoma, and diabetic retinopathy [1,2,3,4], and involve endothelial dysfunction and impaired endothelium-dependent vasodilatation [5,6,7]. Nitric oxide (NO), prostaglandins, and endothelium-dependent hyperpolarization (EDH) mediate endothelium-dependent relaxation [8,9,10], and activation of Ca^2+^-activated potassium channels of small (SKCa, subtype SKCa3 a.k.a KCa2.3) and intermediate (IKCa, a.k.a. KCa3.1) conductance are involved in the activation of these signal pathways [11,12]. Prostaglandin-mediated endothelium-dependent relaxation is of less significance in retinal arterioles [13,14,15,16]. In rats, the EDH pathway is involved in endothelium-dependent relaxation in retinal arteries [17], but this is not the case in retinal arterioles from pigs [13] and man [18]. These findings support that NO is the main mediator of endothelium-dependent relaxation in retinal arterioles [19]. We have previously found that the SKCa channel is involved in the bradykinin-induced NO-mediated relaxation of porcine retinal arterioles [13,14]. Concerning the underlying mechanisms, it has been proposed that the activation of endothelial SKCa channels and the resulting membrane hyperpolarization increases the electrochemical driving force for Ca^2+^ entry and the subsequent activation of Ca^2+^-dependent NO formation and release [20,21,22]. Moreover, endothelial cell hyperpolarization may also decrease superoxide formation by decreasing the activity of NAPDH oxidase [23]. However, in intact vascular preparations, activators of SKCa channels, such as 6,7-Dichloro-1H-indole-2,3-dione 3-oxime (NS309) and cyclohexyl-[2-(3,5-dimethyl-pyrazol-1-yl)-6-methyl-pyrimidin-4-yl]-amine (CyPPA), relax porcine retinal arterioles by a mechanism, which to a large degree are independent of changes in endothelial cell Ca^2+^ [14]. These findings suggest that other mechanisms contribute to the relaxations induced by the activation of SKCa channels in porcine retinal arterioles.

Regulation of endothelial l-arginine uptake has been proposed to play a role for endothelial NO release and blood flow regulation in vivo [24]. This is also supported by the observations that l-arginine uptake, rather than the intracellular l-arginine concentration, is important for NO production in endothelial cells [25]. Indeed, in isolated rabbit eyes, administration of l-arginine decreases ocular vascular tone [26], supporting a major role for l-arginine uptake and NO release in endothelium-dependent relaxation in retinal arterioles. 

The majority of the l-arginine uptake in endothelial cells occurs by the Na^+^-independent (y^+^ system) cationic amino acid transporter (CAT) [27,28]. The l-arginine transport by the y^+^ system appears to depend on the membrane potential, where hyperpolarization increases and depolarization decreases l-arginine uptake [29,30]. Based on these findings, we hypothesized that hyperpolarization caused by activating SKCa channels increases l-arginine uptake through the y^+^ transporter system and enhances NO-mediated relaxation.

To address the hypothesis, we performed measurements of ^3^H-l-arginine uptake in the absence and the presence of SKCa and IKCa channel blockers and of the CAT system in isolated rat aortic valve endothelial cells, which served as an in situ endothelial cell preparation avoiding l-arginine uptake in other cell types. We performed immunohistochemistry in porcine retinal arterioles to reveal the localization of endothelial NO synthase (eNOS), SKCa3, and CAT-1 protein. In isolated porcine retinal arterioles, we studied the effect of l-arginine in the absence and the presence of inhibitors of the CAT system on relaxation induced by bradykinin and CyPPA, an activator of SKCa3 channels [31]. Our findings suggest that SKCa channel activity regulates L-arginine uptake through the y^+^ transporter system.

## 2. Results

### 2.1. l-arginine Uptake in Rat Valve Endothelial Cells

**Figure 1:**
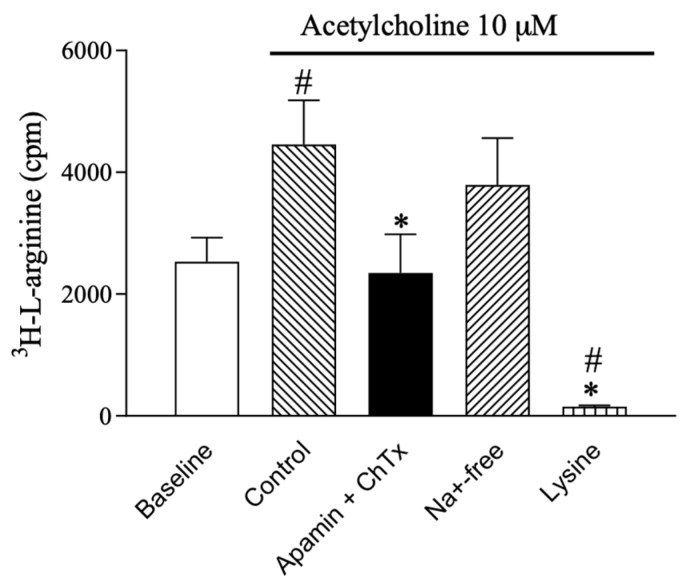
^3^H-l-arginine uptake in rat aortic valve endothelial cells in the absence and presence of the combination of apamin (0.5 µM), a SKCa channel blocker, and charybdotoxin (ChTX, 0.1 µM), an IKCa channel blocker, and of l-lysine (600 µM), both inhibiting l-arginine uptake through the CAT system, and of low Na^+^-PSS which inhibits L-arginine uptake through the Na^+^-dependent (Bo,+) CAT system. Data are means ± S.E.M. (*n* = 6). One-way ANOVA. # *p* < 0.05 vs. baseline. * *p* < 0.05 vs. control.

To address the role of SKCa and IKCa channels in l-arginine uptake, ^3^H-l-arginine uptake was investigated in aortic valve endothelial cells. Baseline uptake of ^3^H-l-arginine was 2532 ± 394 cpm (*n* = 6). This uptake increased to 4458 ± 726 cpm (*n* = 6) in the presence of 10 µM acetylcholine. Uptake was abolished by the combination of SKCa- and IKCa channel blockers, apamin (0.5 µM) and ChTX (0.1 µM) (2345 ± 632 cpm, *n* = 6). In contrast, replacing normal PSS with low Na^+^-containing PSS to inhibit the Bo,+ transporter system had no effect on ^3^H-l-arginine uptake (3792 ± 772 cpm), whereas l-lysine (600 µM) that inhibits l-arginine uptake by the y^+^ transporter system, virtually abolished ^3^H-l-arginine uptake (151 ± 23 cpm) (Figure 1).

### 2.2. Localization of eNOS, SKCa3 and CAT-1 Protein

**Figure 2:**
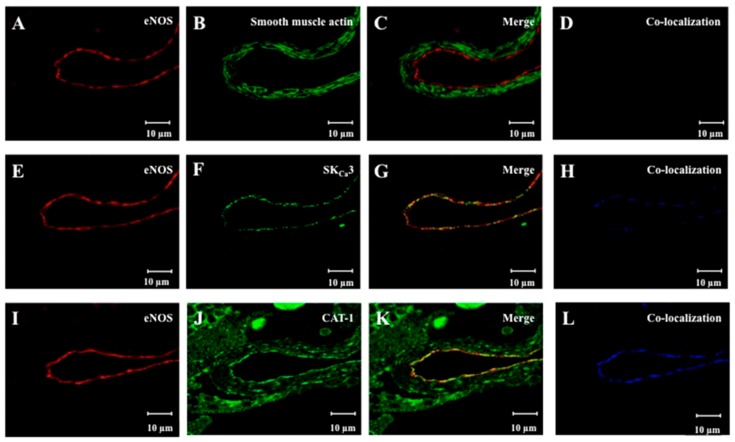
Representative images showing immunoreaction for (**A**) eNOS, (**B**) smooth muscle actin, and (**C**) merged immunoreaction for eNOS and smooth muscle actin, and (**D**) no co-localization of eNOS and smooth muscle actin. Immunoreaction for (**E**) eNOS, (**F**) SKCa3, and (**G**) merged immunoreaction for eNOS and SKCa3, and (**H**) co-localization in blue of eNOS and SKCa3. Immunoreaction for (**I**) eNOS, (**J**) CAT-1, and (**K**) merged immunoreaction for eNOS and CAT-1, and (**L**) co-localization in blue of eNOS and CAT-1.

To evaluate the localization of eNOS, SKCa3 and CAT-1 protein in retinal arterioles, the vascular segments were fixed with surrounding retinal tissue, and eNOS, smooth muscle actin, SKCa3, and CAT-1 were immune-labeled (Figure 2).

eNOS and SKCa3 immunoreactions were observed in the vascular endothelium (Figure 2E–G), but not in the vascular smooth muscle layer (Figure 2C). Immunoreactions for smooth muscle actin were observed in the vascular smooth muscle layer, but not in the vascular endothelium. Furthermore, immunoreactions for CAT-1 were found both in the vascular endothelium and the smooth muscle cells (Figure 2J,K). eNOS was found to be co-localized with SKCa3, and CAT-1 in the endothelium (Figure 2H,L). 

### 2.3. Pharmacological Activation of SKCa Channels in Porcine Arterial Endothelial Cells

**Figure 3:**
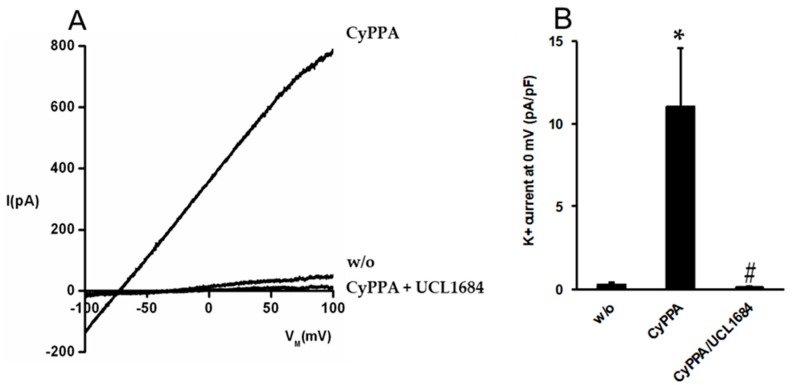
Activation of SKCa currents in porcine arterial endothelial cells by CyPPA. (**A**) Representative whole-cell currents in the absence (w/o) and in the presence of CyPPA (10 µM) as well as inhibition of the CyPPA-activated currents by the SKCa blocker, UCL-1684 (1 µM). (**B**) Average data are given as means ± S.E.M. (CyPPA, *n* = 4; UCl1684, *n* = 3); * *p* < 0.05 *vs*. w/o; # *p* < 0.05 *vs*. CyPPA.

Based on cell lines expressing SKCa channels, CyPPA is considered an activator of the SKCa-subtypes, SKCa2 and SKCa3 [31]. To investigate whether CyPPA activates SKCa currents in primary endothelial cells, we performed patch clamp electrophysiology on porcine arterial endothelial cells (Figure 3).

In primary endothelial cells isolated from porcine coronary arteries, CyPPA (10 µM) consistently induced large outward K^+^ currents, shifting the reversal potential towards negative values near the K^+^ equilibrium potential. The apamin-mimicking non-peptidic blocker of SKCa channels, UCL1684 [32], fully blocked this current (Figure 3B). These data demonstrate that CyPPA is capable of opening SKCa channels in porcine primary endothelial cells. 

### 2.4. Effect of l-arginine on Endothelium-Dependent Relaxation

**Figure 4:**
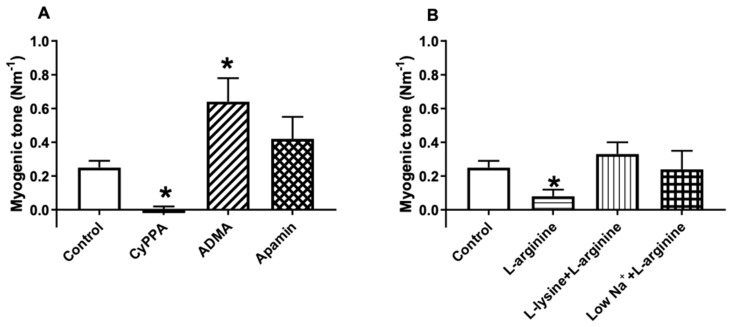
Pharmacological modulation of myogenic tone in porcine retinal arteries by (**A**) an opener of SKCa channels, CyPPA (6 µM, *n* = 6), an inhibitor of NO synthase, asymmetric dimethylarginine (ADMA, 100 µM, *n* = 7), and a blocker of SKCa channels, apamin (0.5 µM, *n* = 8) and by (**B**) substrate for NO synthase, l-arginine (100 µM, *n* = 9), and l-arginine in the presence of blockers of the y^+^ transporter, l-lysine (600 µM, *n* = 9) or low Na^+^-PSS (*n* = 9). Data are means ± S.E.M. * *p* < 0.05 *vs*. control.

Previous studies have shown that endothelium-derived NO may play an important role in basal tone of retinal arterioles [13]. To investigate the role of SKCa channels and l-arginine for basal endothelium-derived NO release, the effect on basal tone was studied. The porcine retinal arterioles developed myogenic tone (*n* = 9), which was increased in the presence of ADMA (300 µM, *n* = 7), an inhibitor of NO synthase, suggesting basal release of NO. An opener of SKCa channels, CyPPA (6 µM, *n* = 6) decreased myogenic tone, while it remained unchanged in the presence of apamin (0.5 µM, *n* = 8) (Figure 4A). The myogenic tone was decreased in the presence of l-arginine (100 µM, *n* = 9) alone, while it was unchanged in the presence of the combination of l-lysine (600 µM) (*n* = 9) and l-arginine (100 µM) and of low Na^+^ (*n* = 9) plus l-arginine (100 µM) (Figure 4B). These findings suggest that both l-arginine and the activation of SKCa channels by CyPPA increase basal release of NO in retinal arteries. 

**Figure A1:**
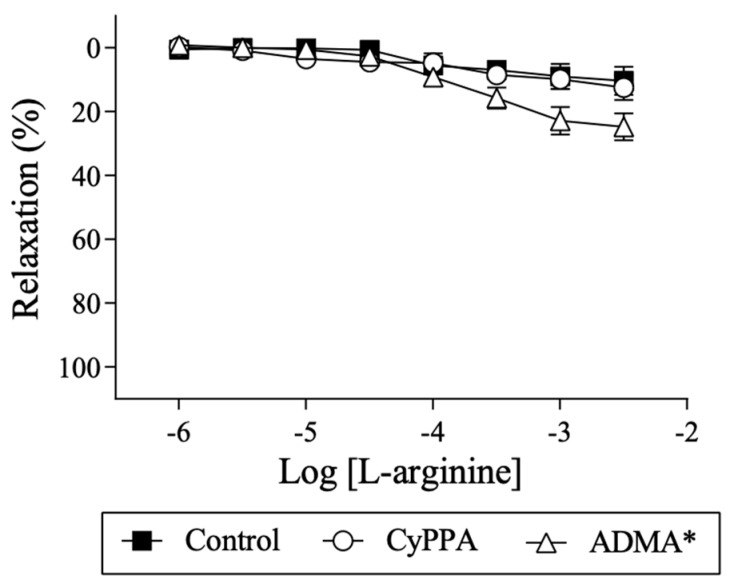
l-arginine-induced relaxation in the absence and presence of CyPPA (6 µM), an opener of SKCa2 and SKCa3 channels, or ADMA (300 µM), an inhibitor of NO synthase, in U46619-contracted porcine retinal arterioles. Data are means ± S.E.M. (*n* = 5–7). Two-way ANOVA. * *p* < 0.05 *vs*. control.

In porcine retinal arterioles contracted with U46619 (0.1 µM), l-arginine (1 µM–3 mM) alone (*n* = 5) or with CyPPA (6 µM) (*n* = 5) did not induce relaxation (Figure A1). In the presence of the inhibitor of eNOS, ADMA (300 µM) (*n* = 7), l-arginine induced small relaxations with a maximum of 25 ± 4% (*n* = 7) at 3 mM l-arginine (Figure A1). These findings suggest that l-arginine in the absence and the presence of activation of SKCa channels are not involved in endothelium-derived modulation of agonist-induced contraction. However, in the presence of the inhibitor of eNOS, ADMA, l-arginine, by competition, is able to reverse the inhibitory effect of ADMA on eNOS-derived NO. 

**Figure 5:**
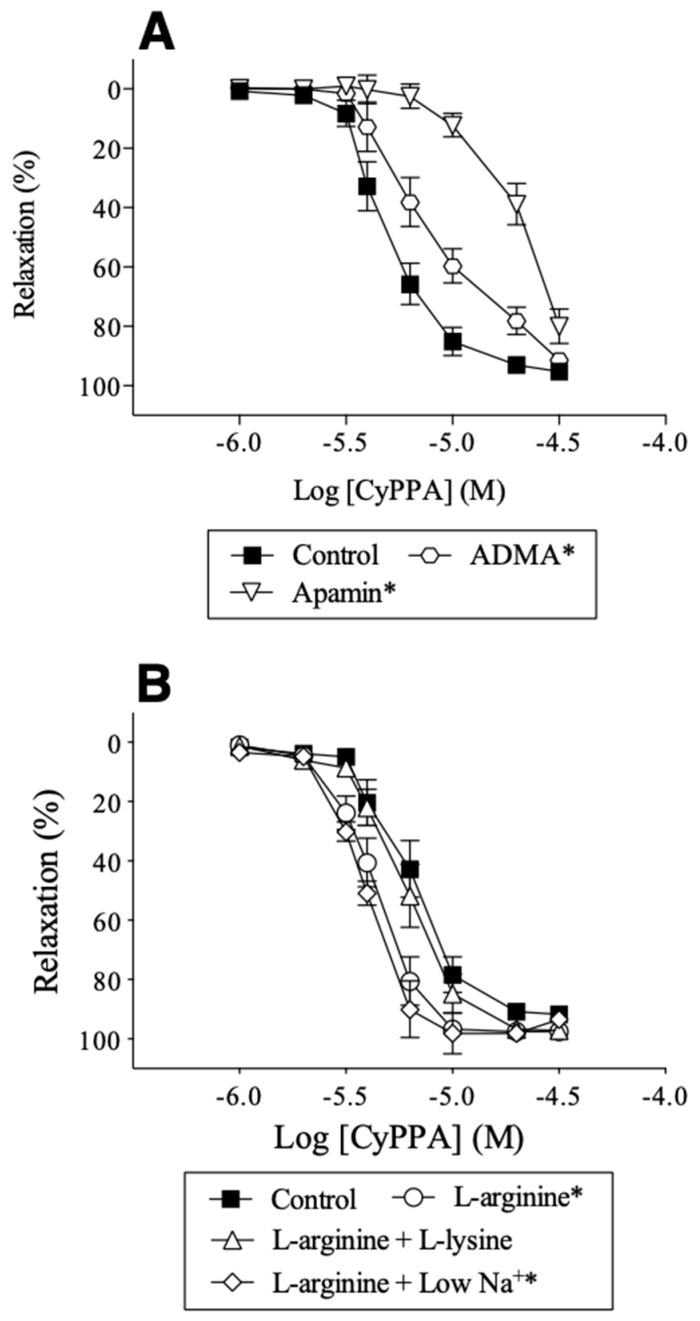
Modulation of CyPPA-induced relaxation in porcine retinal arterioles by (**A**) ADMA (300 µM), an inhibitor of NO synthase, apamin (0.5 µM), a blocker of SKCa channels, by (**B**) l-arginine (100 µM), l-lysine (600 µM), and low Na^+^-PSS. Data are means ± S.E.M. (*n* = 7–9). Two-way ANOVA. * *p* < 0.05 *vs*. control.

The opener of SKCa channels, CyPPA (1 µM–30 µM) induced concentrations-dependent relaxations (pD_2_: 5.18 ± 0.03, *n* = 8) in porcine retinal arterioles. In the presence of ADMA (300 µM) or apamin (0.5 µM), concentration-response curves for CyPPA were significantly rightward shifted with pD_2_–values of, respectively, 4.96 ± 0.04 (*n* = 7) and 4.66 ± 0.08 (*n* = 8, *p* < 0.05) suggesting that the opening of SKCa channels causes relaxation through a mechanism involving NO. In the presence of l-arginine (100 µM), concentration-response curves for CyPPA were leftward shifted with pD_2_ values of 5.36 ± 0.02 (*p* < 0.05 versus control, *n* = 9). This enhanced relaxation was abolished by inhibiting the l-arginine uptake through the y^+^ transporter system with l-lysine (600 µM) (pD_2_: 5.22 ± 0.02, *n* = 9), but not when normal PSS was replaced with low Na^+^ containing PSS to inhibit the l-arginine uptake through the Bo,+ transporter system (pD_2_: 5.41 ± 0.01, *n* = 9) (Figure 5). These findings suggest that increasing extracellular l-arginine via the y^+^ transporter system contributes to NO-mediated relaxation induced by pharmacological activation of SKCa channels.

**Figure A2:**
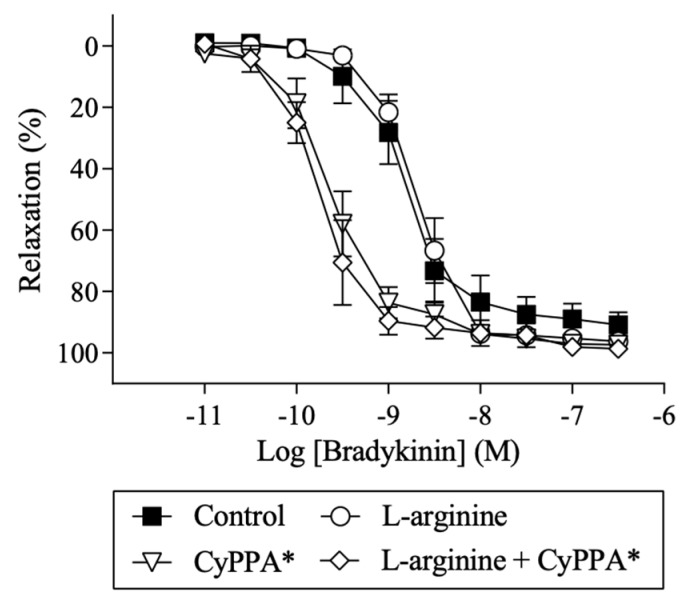
Bradykinin-induced relaxation in the absence and presence of l-arginine (100 µM) and CyPPA (6 µM), an activator of SKCa2-3 channels, in U46619-contracted porcine retinal arterioles. Data are means ± S.E.M. (*n* = 6–7). Two-way ANOVA. * *p* < 0.05 *vs*. control.

Bradykinin induces relaxations in porcine retinal arteries via B_2_ receptor-signaling. These relaxations can be blocked by inhibitors of eNOS and cyclooxygenase [13]. In the present study, bradykinin (0.01 nM–0.3 µM) induced concentration-dependent relaxations (pD_2_: 8.72 ± 0.07, *n* = 6) (Figure A2). In the presence of CyPPA (6 µM), these relaxations were enhanced (pD_2_: 9.56 ± 0.05, *n* = 7). In contrast, l-arginine (100 µM) did not change bradykinin-induced relaxations neither alone (pD_2_: 8.67 ± 0.03, *n* = 6) nor in the presence of CyPPA (pD_2_: 9.71 ± 0.05, *n* = 6) (Figure A2). These findings suggest that the opening of SKCa channels enhances bradykinin relaxation, while extracellular L-arginine does not. 

## 3. Discussion

Our results suggest that in porcine retinal arterioles, eNOS, SKCa3, and CAT-1 protein are co-localized in the vascular endothelium, and that l-arginine enhances NO mediated relaxation induced by activating SKCa channels, an effect mediated by the l-arginine uptake through the y^+^ transporter system. Supporting these findings, acetylcholine-induced ^3^H-l-arginine uptake in rat aortic valve endothelial cells was abolished by blocking the SKCa and IKCa channels or the y^+^ transporter system (Figure 1).

Previous studies report that the majority of eNOS is located in the membrane fraction of endothelial cells, with only a small fraction localized in the cytosol. More specifically, eNOS is localized in caveolae of the endothelial plasma membranes [33,34] and the enzymatic activity of eNOS is primarily particulate and found in caveolar rich parts of the membrane [35]. In electron microscopy, we have observed that SKCa3 channels are expressed in the apical and lateral membrane of endothelial cells in the tissue [36]. Moreover, SKCa3 channels in isolated endothelial cells is in close proximity to caveolin-1, eNOS, and G-protein coupled receptors, such as the bradykinin receptor [37]. These results suggest that SKCa3 and eNOS may co-localize within caveolae of the endothelial cells. For the CAT systems, which are responsible for the uptake of extracellular L-arginine, five transporter proteins have been characterized (CAT-1, CAT-2A, CAT-2B, CAT-3, and CAT-4) [27], and they are categorized into two classes based on their Na^+^ dependence. The carrier proteins mediating Na^+^-independent transport of cationic amino acids include the systems y^+^, y^+^L, bo,^+^, b^+^, where uptake of cationic amino acids occurs via carrier-mediated passive-facilitated diffusion. The carrier proteins mediating Na^+^-dependent transport of cationic amino acids include the Bo,+ system, where uptake of cationic amino acids is coupled with the plasma membrane Na^+^ electrochemical gradient generated by the Na^+^/K^+^ ATPase [38]. The majority of L-arginine enters endothelial cells through the CAT-1 y+ transporter system [25,27], and immunohistochemistry and immunoprecipitation showed that the CAT-1 exists in a complex with eNOS in plasmalemmal caveolae from porcine pulmonary arterial endothelial cells [39], and interact directly in bovine aortic endothelial cell culture [38]. The L-arginine uptake appears to involve the membrane potential, in which hyperpolarization increases and depolarization decreases the uptake [29,30]. In agreement with these findings, we found that eNOS partly co-localized with SKCa3 and CAT-1 (Figure 2) indicating that these proteins may exist as a microdomain and interact in porcine retinal arteriolar endothelial cells. 

Previous studies have found that relaxation by eNOS activation induced by stretch, isometric contration, cAMP, and isoprenaline, but not acetylcholine, is dependent on extracellular l-arginine in rat pulmonary arteries [40,41]. A possible explanation for this discrepancy between these results may be the co-localization of eNOS and CAT-1 within caveolae of endothelial cell [38]. Stretch, isometric contraction, cAMP, and isoprenaline are dependent on extracellular l-arginine through CAT-1 [42], whereas acetylcholine causes translocation of eNOS into the cytosol allowing access to the cytosolic l-arginine pool [43,44], minimizing extracellular l-arginine dependence. Supporting these findings, this study found that the myogenic tone was reduced by l-arginine and SKCa channel activation, and increased by eNOS inhibition (Figure 4). Moreover, l-lysine and low Na+-PSS, normalized the reduced myogenic tone induced by l-arginine. These results suggest that the NO mediated regulation of myogenic tone may be dependent on extracellular l-arginine and SKCa channel activity. 

A decreased NO bioavailability associated with endothelial dysfunction in cardiovascular diseases may be caused by a limited l-arginine supply [27,44], and many animals studies and clinical studies show that l-arginine supplementation improves endothelium-dependent vasodilatation. However, an equal or perhaps even larger number of studies show that l-arginine supplementation has no effect [44]. This problem is termed the l-arginine paradox, and is based on the observation that an increase in extracellular l-arginine concentration from 0.1 to 10 mM increases NO production, despite that Km for eNOS for l-arginine is approximately 10 μM, and that intracellular l-arginine concentrations range between 100 and 800 μM [27]. One explanation may be that l-arginine is sequestered in pools or compartments that are poorly accessible to eNOS. This leaves the extracellular l-arginine as a regulative substrate for eNOS. Alternatively, changes in the levels of the endogenous inhibitor of eNOS, ADMA, by competition inhibits l-arginine uptake by the y+ transporter. We have previously found that in superior mesenteric arteries from renal hypertensive rats, treatment with l-arginine did not restore NO bioavailability [45]. The present results support this, since l-arginine itself did not induce relaxation and had no effect on bradykinin-induced relaxation of pre-contracted arterioles neither alone nor when CyPPA, an activator of SKCa2-3 channels, enhanced bradykinin-induced relaxation. These results indicate that l-arginine supplementation per se does not improve endothelium-dependent vasodilatation in the presence of a vasoconstrictor. However, in the presence of ADMA, l-arginine induced relaxation supporting that ADMA and l-arginine compete for uptake, indicating that increasing concentrations of l-arginine decreased the eNOS inhibiting effect of ADMA. These findings suggest that l-arginine supplementation may be beneficial when ADMA levels are elevated. 

We have previously reported that activating SKCa channels with CyPPA leads to endothelium-dependent relaxation that is only mediated by NO [14]. In this study, we found l-arginine enhanced CyPPA-induced relaxations which were blocked by the y+ transport inhibitor, l-lysine. Moreover, the patch clamp experiments in the present study provide direct evidence that CyPPA opens endothelial SKCa channels in porcine primary endothelial cells. Thus, our findings suggest that the activation of SKCa channels through endothelial cell hyperpolarization leads to increased uptake of l-arginine through the y^+^ transporter system and this is followed by increased NO production.

In conclusion, in porcine retinal arterioles, eNOS, SKCa3, and CAT-1 proteins were localized in the vascular endothelium. l-arginine increased NO mediated relaxation induced by activation of SKCa channels by a mechanism sensitive to inhibition of the y^+^ transporter system. Moreover, in rat aortic valve endothelial cells acetylcholine-induced ^3^H-l-arginine uptake in rat aortic valve endothelial cells was abolished by blocking the SKCa and IKCa channels and the y^+^ transporter system. Taken together, these results suggest that SKCa channel activity regulates l-arginine uptake through the y^+^ transporter system. From a clinical perspective, we propose that l-arginine supplementation alone may not necessarily prevent or restore endothelial dysfunction in cardiovascular disease. In fact, NO donors for instance nitroglycerin do not dilate retinal arteries in patients [46]. Therefore, in patients with retinal diseases, such as age-related macular degeneration, primary open angle glaucoma, and diabetic retinopathy [1,2,3,4], further investigation must clarify whether it is beneficial to combine endothelial SKCa channel activators with l-arginine supplementation to increase the l-arginine uptake and enhance NO mediated relaxation, and hence increase retinal blood flow. 

## 4. Materials and Methods

All experiments conformed to guidelines from the European Convention for the Protection of Vertebrate Animals used for Experimental and other Scientific Purposes and were approved by and conducted with permission from Danish Institutional Animal Care and Use Committee, the Danish Ministry of Environment and Food (#2011/561-2011 from 21 April 2011 and #2014-15-2934-01059 from 19 May 2014).

### 4.1. l-arginine Uptake

In human umbilical vein endothelial cells (HUVECs), the NOS expression decreases markedly from freshly isolated cells to cultured endothelial cells [47]. Therefore, ^3^H-l-arginine uptake was intended in isolated small arteries, but the method did not allow to distinguish l-arginine uptake in vessels with and without endothelium. Therefore, to investigate l-arginine uptake in only in situ endothelial cells, fresh aortic mitral valves from the rat heart were isolated and incubated in physiological saline solution (PSS, see composition below) in an Eppendorf vial. To measure the time-dependent uptake of l-arginine, 20 nM ^3^H-l-arginine was added to the vial oxygenated with a gas mixture of 5% CO_2_, 21% O_2_, and 74% N_2,_ and kept in a bath heated to 37 °C. After 5, 10, and 20 min, the valves were transferred to a plastic tube containing scintillation liquid, and the β emission was measured in a scintillation counter (Model: LS6000, Beckman Coulter Inc., Brea, CA, USA). To investigate the role of potassium channels for the l-arginine uptake, the valves were incubated with acetylcholine (10 µM) for 10 min in the absence or the presence of apamin (0.5 µM) plus charybdotoxin (0.1 µM), low sodium PSS, or low Na^+^-PSS with l-lysine (600 µM) to block the y+ transporter. 

### 4.2. Isolation of Pig Eyes

From a local abattoir (Horsens, Denmark), eyes were obtained from 6 month-old pigs with a weight of 85–90 kg. Immediately after the pigs had been exposed to carbon dioxide (CO_2_) and sacrificed by exsanguination, one eye was removed from each animal and placed in a container with cold (4 °C) physiological saline solution (PSS) of the composition (mM): 4.8 KCl, 1.14 MgSO_4_, 118 NaCl, 25 NaHCO_3_, 5 HEPES, 5.5 glucose, and 1.6 CaCl_2_. The eyes were transported to the laboratory, where they were dissected and the retinas isolated as previously described [14]. The eyes were opened, and the retina was detached from the underlying pigment epithelium. An arteriolar segment with a length of approximately 2 mm was dissected from the retina approximately 1–2 mm from the optic disk.

### 4.3. Immunohistochemistry

Arteriolar segments with surrounding retinal tissue were fixed in cold (4 °C) 4% paraformaldehyde, pH 7.0 for 1 h for immunohistochemical processing. The segments were placed in 8% agar prior to embedding in paraffin. Longitudinal sections of 3 μm were cut on a microtome. The sections were placed on glass slides, heated at 80 °C for 1 h, and de-paraffinized in xylene and decreasing concentrations of ethanol. To evaluate the distribution of SKCa3, eNOS, CAT-1, and smooth muscle actin protein, the sections were rinsed with phosphate buffered saline (PBS: 1.5 M NaCl, 0.5 M NaH_2_PO_4_), transferred to a 10 mM citrate buffer (5.0 mM trisodiumcitrate dehydrate, 5.0 mM disodiumhydrogencitrate, pH 6.0), heated in the microwave for 2 × 7 min and rinsed with PBS. The sections were transferred to 0.2% Triton X solution for 10 min and then rinsed with PBS. In order to block non-specific antibody binding, the sections were incubated with 10% fetal calf serum in PBS with 1% BSA for 20 min. Incubation was done overnight in a humidified chamber at 4 °C with primary antibodies. Antibodies for SKCa3 protein (1:400, Antibodies-online GmbH, Aachen, Germany), eNOS protein (1:50, BD Biosciences, Franklin Lakes, NJ, USA), CAT-1 protein (1:100, SLC7A1, Proteintech Group, Chicago, IL, USA), and smooth muscle actin protein (1:500, Dako, Glostrup, Denmark) were applied in PBS containing 1% bovine serum albumin (BSA). Negative controls for each sample section were without primary antibody. The sections were rinsed with PBS. They were then incubated for 1 h at room temperature with a secondary antibody, goat anti-rabbit IgG coupled to Alexa 488 (dilution 1:2000, Molecular Probes Inc., Eugene, OR, USA) or goat anti-mouse IgG coupled to Alexa 633 (dilution 1:2000, Molecular Probes Inc., Eugene, OR, USA) with PBS containing 1% BSA for 1 h followed by rinsing with PBS. Finally, a cover slip was mounted after addition of antifade solution (Bio-Rad Laboratories Ltd., Hemel Hempstead, UK). The preparations were analyzed on an inverted confocal microscopy (LSM 510 Meta, Carl Zeiss Inc, Oberkochen, Germany) equipped with an oil immersion objective (C-Aprochromat 63 × NA = 0.75) excited at a wavelength of 488 or 633 nm and analyzed using the Zeiss Zen Image Browser software program (Zeiss Inc, Oberkochen, Germany).

### 4.4. Patch Clamp Experiments in Endothelial Cells

Porcine arterial endothelial cells were isolated as described previously [48]. In brief, porcine coronary arteries were cut open longitudinally and incubated for 30 min in phosphate-buffered saline w/o Ca^2+^/Mg^2+^ containing trypsin/EDTA (0.25%/0.02%, *w/v*). Thereafter, the luminal surface was gently scrapped with the tip of a 100 μL pipette tip. Detached endothelial cells were aspirated and plated on cover slips in Modified Eagle Medium supplemented with 10% newborn calf serum and penicillin/streptomycin (all from Biochrom KG, Berlin, Germany). Whole cell currents were recorded with an EPC10-USB patch-clamp amplifier and the PatchmasterTM software (HEKA Electronics, Germany) using voltage-ramps (−100 to 100 mV, 1 sec, filter 1000 Hz). K^+^ outward currents were quantified at a potential of 0 mV before (w/o), after addition of CyPPA, and after addition of UCL1684 (both from Tocris Bioscience). The KCl-pipette solution was composed of (in mM): 140 KCl, 1 MgCl_2_, 2 EGTA, 1.71 CaCl_2_ (1 μM [Ca^2+^]_free_), and 5 HEPES (adjusted to pH 7.2 with KOH). The bath solutions was composed of (in mM): 140 NaCl, 5 KCl, 1 MgSO_4_, 1 CaCl_2_, 10 glucose and 10 HEPES (adjusted to pH 7.4 with NaOH).

### 4.5. Functional Studies in Porcine Retinal Arterioles

An isolated arteriolar segment was transferred to a chamber of a dual wire myograph system (model 410A, Danish MyoTechnology, Aarhus, Denmark) for isometric tension recording. For recording the system was coupled to the Chart5 software program (ADInstruments Ltd., Oxfordshire, UK). Each arteriole was mounted in the myograph using two 25 µm tungsten wires and was allowed to equilibrate for 30 min at 37 °C in PSS bubbled with artificial air of the following composition: 5% CO_2_, 21% O_2_, and 74% N_2_. Subsequently, the arterial segments were stretched in PSS without CaCl_2_ (PSS0.0) to an internal circumference corresponding to 94% of the tone at a transmural pressure of 70 mmHg [49]. After setting the passive tension, PSS0.0 was replaced with PSS, and the segment was allowed to equilibrate for another 30 min. The internal diameter of the arterioles used for functional studies averaged 118 ± 2 µm (*n* = 79).

To test smooth muscle cell viability potassium-rich PSS (KPSS) was added to test contraction by smooth muscle cell depolarization. KPSS was PSS, where equimolar concentration of NaCl was replaced with KCl giving a concentration of 60 mM K+. Moreover, 9, 11-dideoxy-11a, 9a-epoxymethano-prostaglandin F_2α_ (U46619, 0.1 µM) was added to examine contraction induced by activation of the thromboxane A_2_ receptor. The arterioles were discarded, if U46619 contraction was less than 0.25 Nm^−1^. To test endothelial function, bradykinin (0.03 µM) was added in U46619 (0.1 µM)-contracted arterioles. The arteries were discarded if bradykinin relaxation was less than 50% [13]. 

After control of viability, the segments were contracted with U46619 (0.1 µM) and concentration-response curves were obtained by cumulative additions of L-arginine (1 µM–3 mM) bradykinin (0.01 nM–0.3 µM), and CyPPA (1–30 µM). To investigate the contribution from NO synthase in the concentration-response experiments, the segments were incubated with the NO synthase inhibitor, asymmetric dimethylarginine (ADMA, 300 µM) [45] or l-arginine (100 µM) [50] 30 min prior to contraction. To investigate the role of Na^+^-dependent (Bo,+) and independent (y+) CAT system, the preparations were incubated with l-lysine (600 µM) to inhibit l-arginine uptake through the CAT systems [40,41] or low Na^+^-PSS to inhibit the Na^+^-dependent (Bo,+) CAT system, where NaCl was replaced with choline chloride. To investigate the effect of activating SKCa channels on bradykinin-induced relaxation, CyPPA (6 µM) was added to the organ bath after the segments were contracted with U46619 (0.1 µM) and then concentration-response curves for l-arginine or bradykinin were obtained [13].

### 4.6. Drugs and Solutions

Xylene, Meyer’s haematoxylin and eosin for histological stainings were obtained from Bie & Berntsen, Herlev, Denmark. Fetal calf serum was purchased at Biochrom, Berlin, Germany. Triton X and BSA were purchased at Sigma Aldrich, St. Louis, MO, USA. U46619, ADMA, bradykinin, l-arginine, and l-lysine were from Sigma Aldrich, St. Louis, MO, USA. CyPPA (cyclohexyl-[2-(3,5-dimethyl-pyrazol-1-yl)-6-methyl-pyrimidin-4-yl]-amine) was a donation from Dr. Søren-Peter Olesen, Neurosearch A/S, Ballerup, Denmark. U46619 was dissolved in ethanol. CyPPA was dissolved in DMSO (99%), and bradykinin in distilled water. Bradykinin was prepared in 2% albumin-coated Eppendorf tubes. 

### 4.7. Data and Statistical Analysis

The Zeiss Zen Image Browser software program (Zeiss Inc, Oberkochen, Germany) was used for the immunolabeling and co-localization analysis. Based on these analyses, weighted co-localization coefficients were calculated. The sum of intensities of co-localizing pixels in channel 1 or 2, respectively, was compared to the overall sum of pixel intensities above threshold. Value range 0-1 (0: no co-localization, 1: all pixels co-localize). Bright pixels were considered to contribute more than faint pixels.

For ^3^H-l-arginine uptake experiments the measurements are given as counts per min (cpm). For functional studies, myogenic tone was calculated as the change in tension after the normalization procedure and after 30 min of incubation with inhibitors. In the concentration-response experiments, relaxations were calculated as the tension in the peak response after each addition of bradykinin, while for l-arginine or CyPPA the stable tension after each addition were expressed as percentage of the active tension. The active tone was defined as the level of contraction after the addition of U46619 subtracted by the level of contraction after the passive stretch of the preparations. For bradykinin curves obtained in the presence of CyPPA, the active tone was defined as the level of contraction after addition of U46619 and CyPPA subtracted by the level of contraction after passive stretch of the preparations. 

All data are means ± S.E.M. with a significance level of *p* < 0.05. *n* represents the number of segments from individual animals. Concentration-response curves were compared by use of two-way ANOVA. One-way ANOVA followed by Bonferroni post-hoc test was used for evaluation of differences in ^3^H-l-arginine uptake and in myogenic tone experiments. All data were analyzed using GraphPad Prism 7.0 software (GraphPad software Inc., La Jolla, CA, USA). 

## Abbreviations

ADMA	Asymmetric dimethylarginine
CAT-1	cationic amino acid transporter
CyPPA	cyclohexyl-[2-(3,5-dimethyl-pyrazol-1-yl)-6-methyl-pyrimidin-4-yl]-amine
EDH	Endothelium-derived hyperpolarization
eNOS	Endothelial nitric oxide synthase
IKCa	Ca^2+^-activated K channels with intermediate conductance (KCa3.1)
NO	Nitric oxide
SKCa	Ca^2+^-activated K channels with small conductance (KCa2.1-3)
UCL1684	Blocker of Ca^2+^-activated K channels (KCa2.1-3)
